# Genome Sequences of Bordetella pertussis Isolates from Outbreaks in Northeastern Mexico

**DOI:** 10.1128/mra.00096-23

**Published:** 2023-04-03

**Authors:** Nadia Rodríguez-Medina, Jessica Gutiérrez-Ferman, Licet Villarreal-Treviño, Ulises Garza-Ramos, Héctor Jesús Maldonado-Garza, José Bárcenas-Walls, Rocío Ortiz-López, Manuel de-la-O-Cavazos, Consuelo Treviño-Garza, María Romelia Ballesteros-Elizondo, Elvira Garza-González

**Affiliations:** a Laboratorio de Resistencia Bacteriana, Instituto Nacional de Salud Pública (INSP), Centro de Investigación Sobre Enfermedades Infecciosas (CISEI), Cuernavaca Morelos, Mexico; b Departamento de Microbiología General, Facultad de Ciencias Biológicas, Universidad Autónoma de Nuevo León, San Nicolás de los Garza, Nuevo León, Mexico; c Facultad de Medicina, Hospital Universitario “Dr. José Eleuterio González,” Universidad Autónoma de Nuevo León, Monterrey, Nuevo León, Mexico; d Unidad de Biología Molecular, Genómica y Secuenciación, Centro de Investigación y Desarrollo en Ciencias de la Salud, Universidad Autónoma de Nuevo León, Monterrey, Nuevo León, Mexico; e Secretaría de Salud de Nuevo León, Monterrey, Nuevo León, Mexico; University of Maryland School of Medicine

## Abstract

Here, we report the draft genome sequences of 4 Bordetella pertussis isolates which correspond to major clones isolated between 2008 and 2014 from two outbreaks in northeastern Mexico. The B. pertussis clinical isolates belong to the *ptxP3* lineage, and they are grouped into two major clusters, defined by the *fimH* allele.

## ANNOUNCEMENT

Bordetella pertussis is the etiologic agent of pertussis, a vaccine-preventable disease (1–6). The current immunization schedule in Mexico includes four doses of acellular vaccine (ACV), followed by a dose of whole-cell vaccine (WCV) (7). It appears that the adaptation of B. pertussis goes beyond changes in the antigens present in the ACV vaccine and involves other genes (8, 9). Evidence points to an increase of pertussis in some countries, including Australia, Canada, the United States, the Netherlands, and Mexico (10–16).

The Secretariat of Health of Nuevo León, Mexico, has an extensive collection of 352 B. pertussis isolates recovered between 2007 and 2014. We previously characterized 113 of the isolates using pulsed-field gel electrophoresis (PFGE) and identified one major clone (A1) and three closely related clones (14). In this study, we performed whole-genome sequencing of four representative clones of B. pertussis circulating in the north of Mexico (Table 1). These colonies were selected for growth on LB agar and then inoculated in LB broth at 37°C with shaking at 200 rpm overnight. Genomic DNA was extracted using the MasterPure complete DNA and RNA purification kit (MC85200; Epicentre). Illumina MiSeq libraries were constructed using the Nextera XT sample prep kit (Illumina, Inc., San Diego, CA, USA) and sequenced on standard flow cells in 2 × 150-bp, paired-end format. The Illumina reads were quality checked using FastQC v0.11.8 software (https://www.bioinformatics.babraham.ac.uk/projects/fastqc/) with default parameters and trimmed using Trim Galore v0.6.4 (https://www.bioinformatics.babraham.ac.uk/projects/trim_galore/). *De novo* assembly of the B. pertussis Illumina reads was performed using the SPAdes v3.6.0 genome assembler (17). The contigs generated by the genome assembly of B. pertussis isolates 224-8, 601-9, 781-14, and 620-12 are shown in Table 1. All genomes were annotated using the National Center for Biotechnology Information (NCBI) Prokaryotic Genome Annotation Pipeline (PGAP) (https://www.ncbi.nlm.nih.gov/genome/annotation_prok/) (18).

**TABLE 1:** Clinical, epidemiological, and genomic data of B. pertussis isolates

Characteristic	Data for isolate:			
	224-8	601-9	620-12	781-14
Clinical and epidemiological data				
Yr	2008	2009	2012	2014
Age	50 yrs	4 mo	1 mo	2 mo
Sex	Female	Female	Female	Male
No. of vaccine doses	Unknown	1	1	1
Symptomatic?	Yes	Yes	Yes	Yes
Province	Apodaca, Nuevo León	Guadalupe, Nuevo León	Monterrey, Nuevo León	Cadereyta, Nuevo León
Genotype	BpA2	BpA1	BpA	BpA3
Genomic data				
Genome size (bp)	3,868,965	3,884,116	3,839,894	3,868,470
G+C content (%)	67.8	67.8	67.9	67.8
*N*_50_ (bp)	19,559	19,430	19,284	20,332
No. of contigs	341	317	301	297
GenBank accession no.	NXFD00000000.1	NXFE00000000.1	NXFF00000000.1	NXFC00000000.1
SRA accession no.	SRX3027547	SRX3027548	SRX3027545	SRX3027546

A BLASTn search showed that the Mexican isolates presented known alleles in the pertussis toxin subunits 1, 2, and 3 (*ptxA*, *ptxB*, and *ptxC*), pertactin (*prn*), and fimbriae (*fimH* and *fimD*). These isolates were found to be distributed within the *ptxP3* lineage in two major clades defined by the *fimH* allele. Isolates 781-14 and 620-12 were found to belong to *ptxP3-ptxA1-fimH1-prn2*, whereas 224-8 and 601-9 were found to belong to *ptxP3-ptxA1-fimH2-prn2*.

### Data availability.

This whole-genome shotgun project has been deposited at GenBank under the accession numbers listed in Table 1 and BioProject accession number PRJNA394744.
